# Exploratory study on the role of *Clonorchis sinensis* infection in promoting cholangiocarcinoma progression

**DOI:** 10.1186/s13071-025-07183-2

**Published:** 2025-12-09

**Authors:** Shitao Li, Yiqi Jiang, Jun Kawanokuchi, Xueling Deng, Yuhong Wu, Yu Chen, Lixia Zeng, Ganghuan Deng, Damian Li, Tingzheng Zhan, Dengyu Liu, Ning Ma, Zeli Tang

**Affiliations:** 1 Department of Cell Biology and Genetics, School of Basic Medical Sciences, Guangxi Medical University, Nanning, China; 2 Division of Health Science, Graduate School of Health Science, Suzuka University of Medical Science, Suzuka, Japan; 3 Schistosomiasis Prevention and Control Department, Hengzhou Center for Disease Control and Prevention, Hengzhou, China; 4 Department of Pathology, Guangxi Medical University Cancer Hospital, Nanning, Guangxi China; 5 Department of Preventive Medicine, School of Public Health, Guangxi Medical University, Nanning, China; 6 Key Laboratory of Longevity and Aging-Related Diseases of Chinese Ministry of Education, Guangxi Medical University, Nanning, China; 7 Key Laboratory of Basic Research On Regional Diseases, Education Department of Guangxi Zhuang Autonomous Region, Guangxi Medical University, Nanning, China; 8 Department of Parasitology, School of Basic Medical Sciences, Guangxi Medical University, Nanning, China

**Keywords:** *Clonorchis sinensis*, Cholangiocarcinoma, CK19, CK7, Metabolic reprogramming

## Abstract

**Background:**

Clonorchiasis, a neglected tropical zoonosis, is caused by chronic infection with *Clonorchis sinensis* (*C. sinensis*). This infection can lead to cholangitis, bile duct epithelial hyperplasia, periductal fibrosis, and cholangiocarcinoma (CCA). However, the underlying carcinogenic mechanisms of CCA remain poorly understood, and there is not a well-developed model for *C. sinensis* CCA.

**Methods:**

A *C. sinensis*-infected Sprague–Dawley rat model, co-treated with *N*-nitrosodimethylamine, was established. The study comprised four groups: negative control (NC), *C. sinensis* infection (CS), *N*-nitrosodimethylamine induction (NDMA), and combined *C. sinensis* infection and *N*-nitrosodimethylamine induction (CSNDMA). Pathological damage to the hepatic ducts was evaluated at 10, 17, and 20 weeks after infection. The expression levels of the relevant genes and proteins were detected using quantitative real-time polymerase chain reaction (qPCR) and immunohistochemistry, respectively. In addition, transcriptome sequencing was carried out on hepatic tissues infected for 20 weeks.

**Results:**

Histopathological analysis using hematoxylin and eosin (H&E) and Masson staining revealed bile duct dilation, inflammatory infiltration, and collagen deposition in the liver tissue of both CS and CSNDMA groups, with the most severe manifestations observed in the CSNDMA group. The CSNDMA group exhibited the earliest onset of CCA, occurring at 10 weeks post infection, with an overall incidence of 63%, peaking at 71% by 20 weeks. The CS group showed a 37% incidence of CCA, while only one case was noted in the NDMA group at 20 weeks. Quantitative PCR demonstrated that *C. sinensis* infection induced upregulation of tumor-related markers in the liver, including *CK19*, *PCNA*, *TP53*, *ITGB1*, and *MMP2*, particularly when co-exposed to *N*-nitrosodimethylamine. Immunohistochemistry detected the significant overexpression of CK19, CK7, and PCNA along bile ducts. Transcriptome sequencing further indicated that *C. sinensis* significantly affected circadian rhythm and metabolic reprogramming in the liver, enriching pathways related to cancer, inflammation, and metabolism, including AMPK, PPAR, mTOR, and FoxO pathways.

**Conclusions:**

*C. sinensis* can effectively promote the pathogenesis of CCA and significantly increase the expression of CCA-related genes (e.g., *CK19* and *CK7*). The inflammation, disrupting circadian rhythms and altering energy metabolism caused by *C. sinensis* infection, may promote the progression of CCA. This study provides a foundational experimental basis for diagnosing and intervening in *C. sinensis*-related CCA.

**Graphical Abstract:**

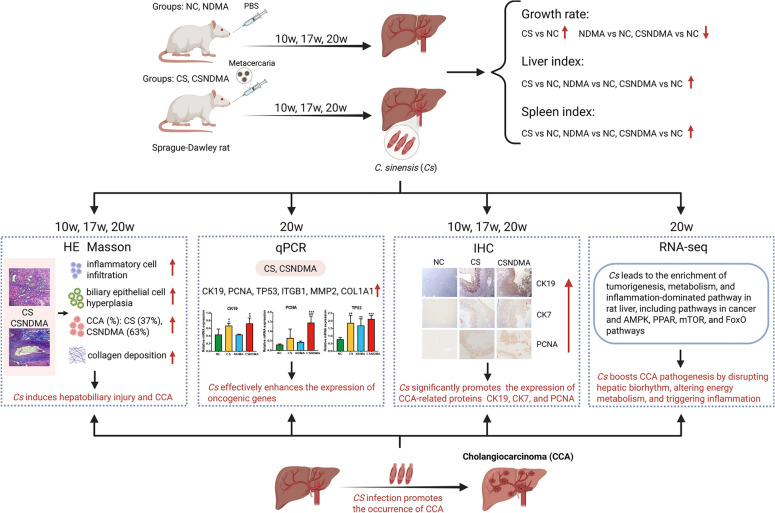

**Supplementary Information:**

The online version contains supplementary material available at 10.1186/s13071-025-07183-2.

## Background

Clonorchiasis, a foodborne parasitic disease caused by *Clonorchis sinensis*, is endemic in several Asian countries, particularly China, South Korea, and Vietnam [1]. In China, this disease predominantly affects the southeastern regions of Guangdong and Guangxi, as well as the northeastern provinces of Heilongjiang and Jilin, with an estimated 10.82 million annual infections [2]. Following the consumption of raw freshwater fish, metacercariae excyst in the duodenum, migrate to the intrahepatic bile ducts, and attach to the biliary epithelium, leading to illness in humans and other definitive hosts [2, 3]. Although early infections often remain asymptomatic, chronic exposure can result in severe hepatobiliary damage, including inflammation, biliary epithelial hyperplasia, periductal fibrosis, and even cholangiocarcinoma (CCA) [2, 4]. The International Agency for Research on Cancer has classified *C. sinensis* as a group 1 carcinogen [5].

CCA is the second most common primary liver malignancy, accounting for approximately 15% of all primary liver cancers [6, 7]. Its incidence and mortality rates have been steadily increasing globally over the past few decades [8]. Identified risk factors for CCA include primary sclerosing cholangitis, chronic hepatitis virus infection, gallstones, and choledochal cystic disease. Notably, liver fluke infections, particularly those caused by *C. sinensis* and *Opisthorchis viverrini*, are major drivers to the development of CCA [2, 9]. The carcinogenic potential of these parasites manifests through three main mechanisms: mechanical injury to the bile duct caused by worm attachment and egg deposition, immunopathological responses to excretory–secretory products, and obstruction-induced biliary stasis leading to subsequent inflammation [2, 10–12]. These synergistic effects drive chronic inflammation, epithelial hyperplasia, and genomic instability, potentially mediated by oxidative stress [2, 4, 13–15], creating a protumorigenic microenvironment.

Despite the established causal link between *C. sinensis* infection and CCA, the precise molecular mechanisms underlying biliary carcinogenesis remain largely uncharacterized. To address this knowledge gap, we established a Sprague–Dawley rat model combining *C. sinensis* infection with *N*-nitrosodimethylamine exposure. Through integrated histopathological evaluation, immunohistochemical analysis, and transcriptomic profiling at multiple time points, this study systematically investigates infection-mediated biliary pathogenesis and its progression to CCA.

## Methods

### Preparation of *C. sinensis* metacercariae

*C. sinensis* metacercariae were collected from naturally infected freshwater fish of *Pseudorasbora parva* in Hengzhou, Guangxi, China. Living metacercariae were obtained by digesting the fish with adjusted artificial gastric juice (pH 2.0, 1.0% NaCl, and 1.0% pepsin). Briefly, minced fish incubated in digestive juice were shaken overnight at 37 °C and 150 rpm. Subsequently, the digestive fluid was filtered through a 60–80 mesh sieve to remove indigestible residues and then rinsed with distilled water until the supernatant in the triangular beaker became clear. Finally, the viable metacercariae were aspirated under an optical microscope and stored in phosphate-buffered saline (PBS) at 4 °C [16].

### Experimental animals and study design

A total of 72 female Sprague–Dawley rats, 5 weeks old and weighing 100–120 g, were procured from the Animal Experiment Center of Guangxi Medical University. The rats were allowed to acclimatize for 1 week and were randomly assigned to four groups: negative control (NC, *n* = 15), *C. sinensis* infection (CS, *n* = 19), *N*-nitrosodimethylamine induction (NDMA, *n* = 18), and *N*-nitrosodimethylamine-induced and *C. sinensis* infection (CSNDMA, *n* = 20). At the onset of the experiment, each rat in the CS and CSNDMA groups was infected with 180 metacercariae via a single gavage. In contrast, the rats in the NC and NDMA groups were administered an equal volume of normal saline. The rats in the NC and CS groups had access to pure drinking water. Those in the NDMA and CSNDMA groups drank water containing 12.5 ppm *N*-nitrosodimethylamine until week 17 of the experiment and then were reverted to normal pure water after that [17]. The rats in each group were weighed every 2 weeks. At 10 and 17 weeks, five to six rats were randomly selected from each group for euthanasia. Given that the condition of the rats in the CSNDMA and NDMA groups deteriorated at 20 weeks, all remaining rats were humanely euthanized at 20 weeks following the start of the experiment.

### Screening for *C. sinensis* eggs in feces

At 6 weeks post-experimental initiation, fecal samples from all groups (NC, CS, NDMA, and CSNDMA) were analyzed using the hydrochloric acid-ether precipitation method [18, 19] to detect *C. sinensis* eggs and assess infection status. Fecal specimens were digested with 3 mL 50% hydrochloric acid in 15 mL centrifuge tubes, followed by 2 mL ether addition and vigorous mixing. Tubes were inverted vigorously and centrifuged (3000 rpm, 3 min), and the supernatant was discarded. Residual liquid was aspirated onto glass slides, covered, and examined under an inverted microscope.

### Pathological analysis of hepatic tissues

Post euthanasia, hepatic tissues from the liver margin, center region, and peribiliary areas were collected, fixed in 4% paraformaldehyde, paraffin-embedded, and sectioned into 4 μm slices. The sections underwent hematoxylin and eosin (H&E) and Masson’s trichrome staining for histopathological evaluation. Hyperplasia was assessed by measuring the length of intrahepatic bile duct glands, while collagen-stained areas were quantified using Image J software (NIH, Bethesda, USA) [20].

The diagnosis of CCA was made on the basis of H&E-stained sections, which identified key malignant features. These features included the presence of irregular, infiltrative tubular or acinar glands of variable sizes, sometimes forming micropapillary or cribriform structures, and lines formed by low-to-moderately differentiated columnar or cuboidal epithelium resembling cholangiocytes. The tumors typically exhibit nodular or infiltrative growth patterns with irregular glandular contours and luminal projections, and often invade the ductal wall and hepatic parenchyma [21–23]. Intrahepatic CCA (iCCA) is categorized into small- and large-duct type. The small-duct type features small cuboidal cells with uniform round nuclei arranged in small tubular or acinar structures, and it often invades hepatic parenchyma nodularly without extensive infiltration. In contrast, the large-duct type comprises large, irregularly dilated glands within abundant fibrous stroma, lined by columnar or cuboidal cells with atypical, hyperchromatic nuclei. This type is characterized by extensive periductal infiltration, vascular and perineural invasion, and associated intraductal dysplasia [8, 24–27]. The entire liver pathological evaluation was conducted blindly.

### Quantitative real‑time PCR (qPCR)

To further determine the molecular mechanism of *C. sinensis* promoting CCA, the mRNA levels of genes closely related to carcinogenesis, including *PCNA*, *CK19*, *TP53*,* ITGB1*, *MMP2*, *COL1A1*, *FADS2*, *COL4A1*, and *TUBB2A* were measured. According to the instructions, the total RNA of 20-week-old rat liver tissue was extracted using the Animal Tissue Total RNA Extraction Kit (TianGen, Beijing, China). The cDNA was generated using TransScript^®^ Uni All-in-One First-Strand cDNA Synthesis SuperMix for qPCR (One-Step gDNA Removal) (TransGen, Beijing, China). qPCR was performed using the StepOnePlus^™^ real-time fluorescent quantitative PCR system (Thermo Fisher Scientific,Waltham, MA, USA). Gene expression levels were normalized to the housekeeping gene β-actin. The primer sequences of the above genes used in the qPCR analysis are provided in Additional File 1: Supplementary Table S1.

### Immunohistochemistry (IHC)

The liver tissues sections underwent standard dewaxing, hydration, and antigen retrieval in 5% urea via 5-min heating. After PBS rinsing, endogenous peroxidase activity was quenched using 3% H_2_O_2_, followed by PBS washing. Nonspecific binding was blocked with 1% skim milk/PBS for 15 min. Primary antibodies for cytokeratin 19 (CK19) (Proteintech Group, Wuhan, China, 1:100), cytokeratin 7 (CK7) (Proteintech Group, Wuhan, China, 1:500), or proliferating cell nuclear antigen (PCNA) (Proteintech Group, Wuhan, China, 1:300) were applied and incubated overnight at 4 °C with gentle shaking. After a routine wash, the sections were treated with a secondary antibody (Vector Laboratories, Newark, California, USA, 1:400) and incubated at room temperature for 45 min, followed by processing with the ABC complex for 60 min. After another PBS wash, color development was achieved using 3,3′-diaminobenzidine. Additionally, CK19- and CK7-stained sections were counterstained with hematoxylin for 30 s. Finally, the slides were washed with pure water, mounted, and observed under an upright microscope (ZEISS, Oberkochen, Germany). They were analyzed using Image J software (NIH, Bethesda, USA).

### RNA sequencing

To evaluate gene expression levels, total RNA was extracted from the liver tissues of three rats per group at 20 weeks using an RNA extraction kit (QIAzol Lysis Reagent, Qiagen, Germany) for both quantitative and qualitative analysis. Transcriptome sequencing analysis was conducted by Shanghai Majorbio Bio-Pharm Technology Co., Ltd., utilizing the NovaSeq X Plus platform to sequence all transcribed mRNAs. And sequencing was performed using an Illumina NovaSeq Reagent Kit for library construction. Quality control of the raw data were executed using Fastp software. Subsequently, HiSat2 was employed to align the obtained data with the reference genome. The RSEM software was then used to quantitatively analyze the expression levels of genes and transcripts. After obtaining the read counts for genes using DESeq2, differential gene expression analysis was conducted to identify differentially expressed genes (DEGs) and further explore their functions. The analysis was conducted on the Majorbio Cloud platform for the DEGs, which encompassed Venn diagram analysis, cluster analysis, expression statistical analysis, and volcano plot analysis. Finally, the public databases Gene Ontology (GO) and Kyoto Encyclopedia of Genes and Genomes (KEGG) were utilized to analyze transcriptional information.

### Statistical analyses

Histological results were expressed as mean ± standard deviation (SD). Data analyses were performed using SPSS 23.0 software (IBM Corp., Armonk, NY, USA). One-way analysis of variance (ANOVA) was used to analyze the histological data, and statistical significance was set at *P* < 0.05. For transcriptomic data, the clean data of each sample reached more than 5.89 Gb, with the number of clean reads exceeding 40,094,820 and the Q30 base percentage being above 94.18%. The DEG analysis was performed using DESeq2 software. The screening criteria for DEGs were set as |log2FC| ≥ 1 and *P* < 0.05. Fisher’s exact test was used for GO and KEGG enrichment analyses. To control for the false-positive rate in the calculations, *P*-values were corrected for multiple tests using the Benjamini–Hochberg method. The significance of gene enrichment in GO and KEGG enrichment analyses was determined on the basis of an adjusted *P*-value of < 0.05.

## Results

### Confirmation of *C. sinensis* infection in rats

The fecal examination results revealed that 6 weeks post infection with *C. sinensis* metacercariae, eggs were detected in the feces of every rat in both the CS and CSNDMA groups, indicating a 100% infection rate, while no eggs were observed in the NC and NDMA groups.

### The influence of *C. sinensis* infection on the body weight and hepatosplenic index of rats

Throughout the experiment, the body weight of the rats in each group exhibited an upward trend (Additional File 1: Supplementary Fig. S1A). The NC group showed the fastest rate of body weight increase, whereas the CSNDMA group showed the slowest rate. Additionally, the body weight of rats in the CS group increased at a faster rate than that in the NDMA group (Additional File 1: Supplementary Fig. S1B). At 10, 17, and 20 weeks, the liver indices of the CS, NDMA, and CSNDMA groups were higher than those of the NC group (Additional File 1: Supplementary Fig. S1C–E). In terms of the spleen index, the CS, NDMA, and CSNDMA groups also displayed an upward trend relative to the NC group (Additional File 1: Supplementary Fig. S1F–H), with the NDMA group showing the most pronounced increase and the most significant difference from the NC group.

### The gross and pathological changes as well as the incidence of CCA in the rat liver induced by *C. sinensis* infection

**Fig. 1:**
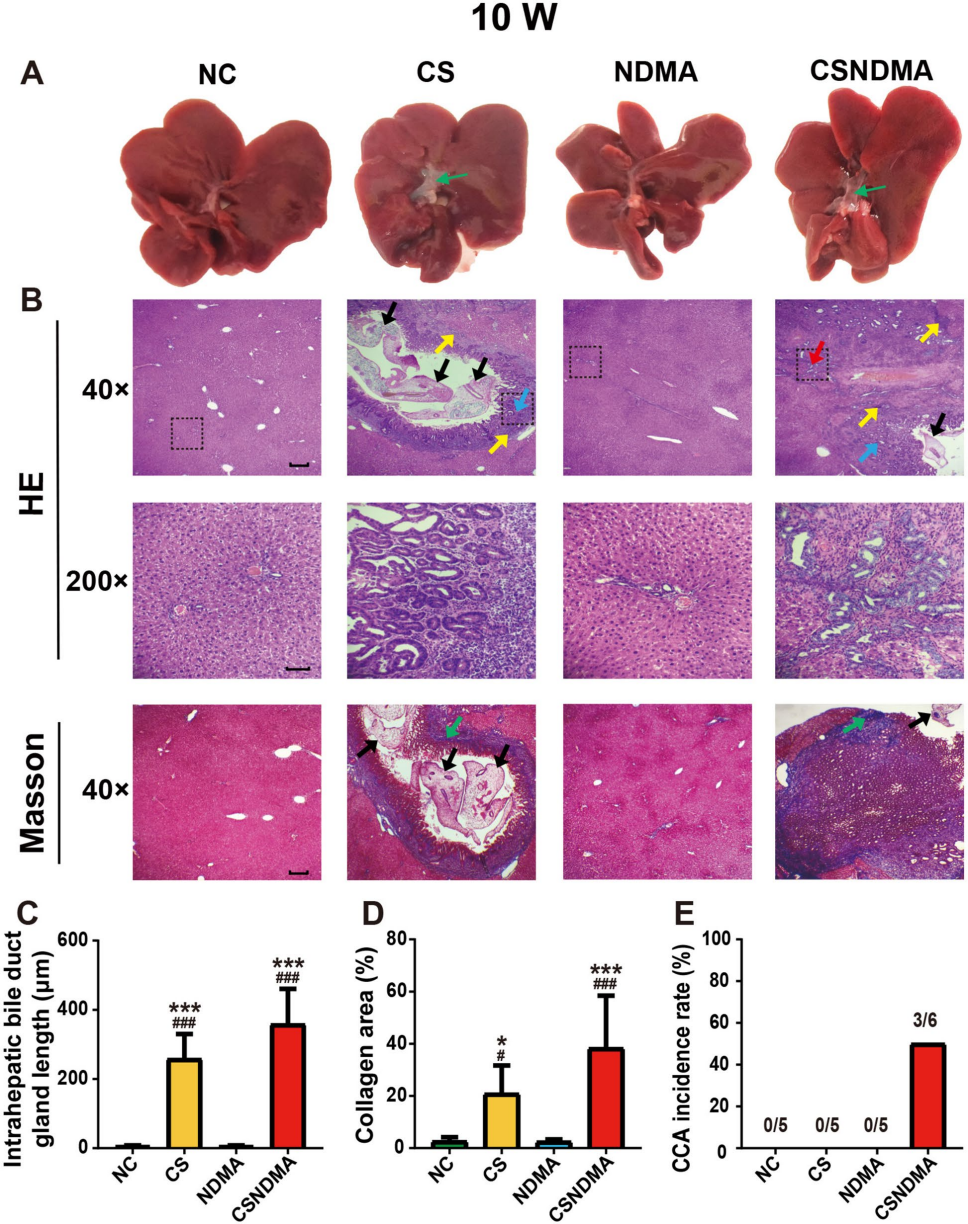
Gross appearance and histopathological changes in the livers of rats infected with *C. sinensis* at 10 weeks. **A** Gross morphology of the liver, with bile duct dilation indicated by thin green arrows. **B** H&E and Masson staining of liver sections. The 200× images correspond to the areas outlined by the dashed boxes in the 40× images. Adult worms (thick black arrows), inflammation (yellow arrows), hyperplasia (blue arrows), CCA (red arrows), and collagen fiber deposition (green arrows) are marked. **C** Intrahepatic bile duct gland length. **D** Percentage of collagen fiber area in liver tissue. **E** Incidence of CCA. Data are presented as mean ± SD. One-way ANOVA analysis of variance was used for statistical analysis. Statistical significance: ^*^P < 0.05 and ^***^P < 0.001 versus NC group; ^#^*P* < 0.05 and ^###^*P* < 0.001 versus NDMA group. Scale bars: 200 μm (40×), 50 μm (200×)

**Fig. 2:**
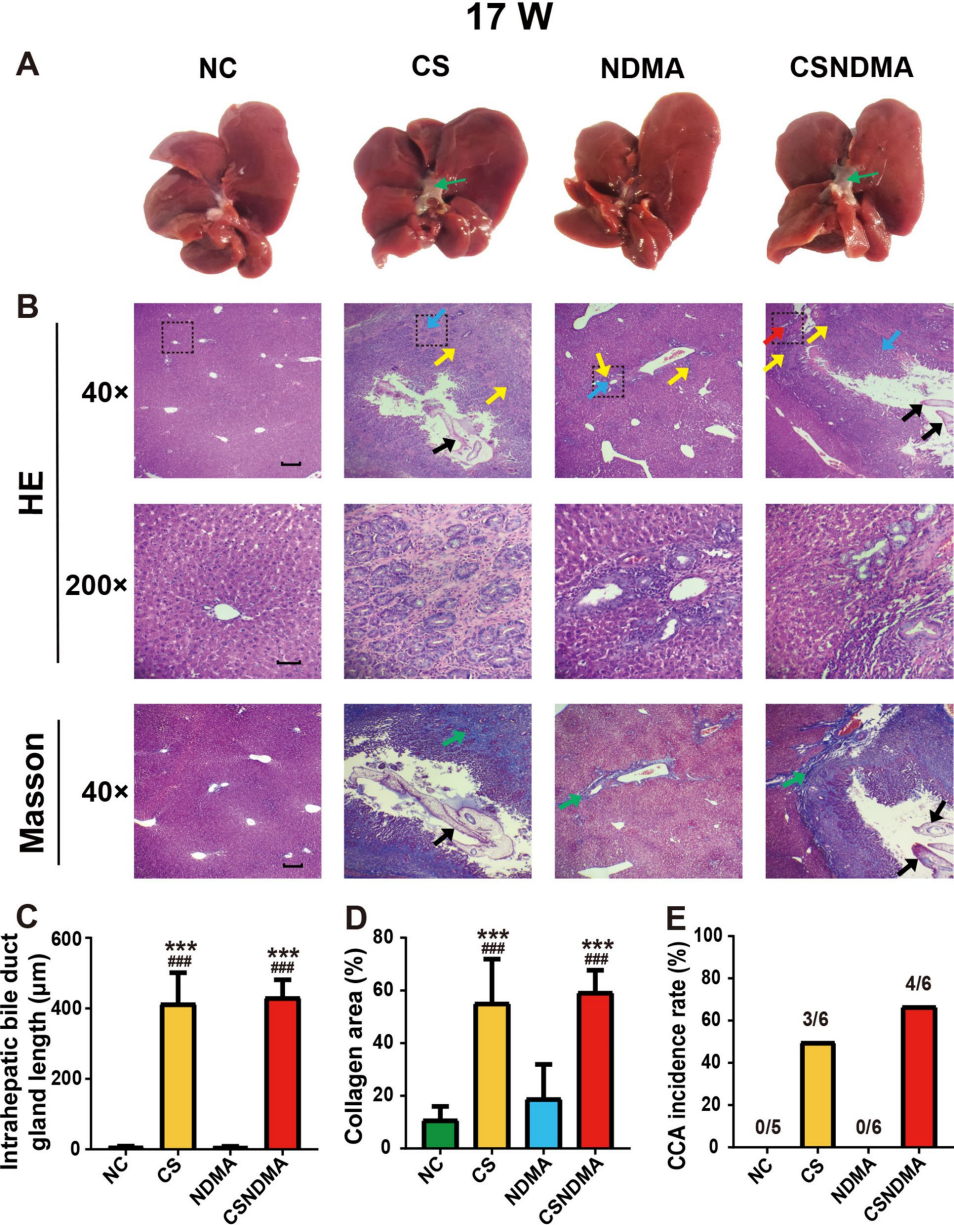
Gross appearance and histopathological changes in the livers of rats infected with *C. sinensis* at 17 weeks. **A** Gross morphology of the liver, with bile duct dilation indicated by thin green arrows. **B** H&E and Masson staining of liver sections. The 200× images correspond to the areas outlined by the dashed boxes in the 40× images. Adult worms (thick black arrows), inflammation (yellow arrows), hyperplasia (blue arrows), CCA (red arrows), and collagen fiber deposition (green arrows) are marked. **C** Intrahepatic bile duct gland length. **D** Percentage of collagen fiber area in liver tissue. **E** Incidence of CCA. Data are presented as mean ± SD. One-way ANOVA analysis of variance was used for statistical analysis. Statistical significance: ^*^P < 0.05 and ^***^P < 0.001 versus NC group; ^#^*P* < 0.05 and ^###^*P* < 0.001 versus NDMA group. Scale bars: 200 μm (40×), 50 μm (200×)

**Fig. 3:**
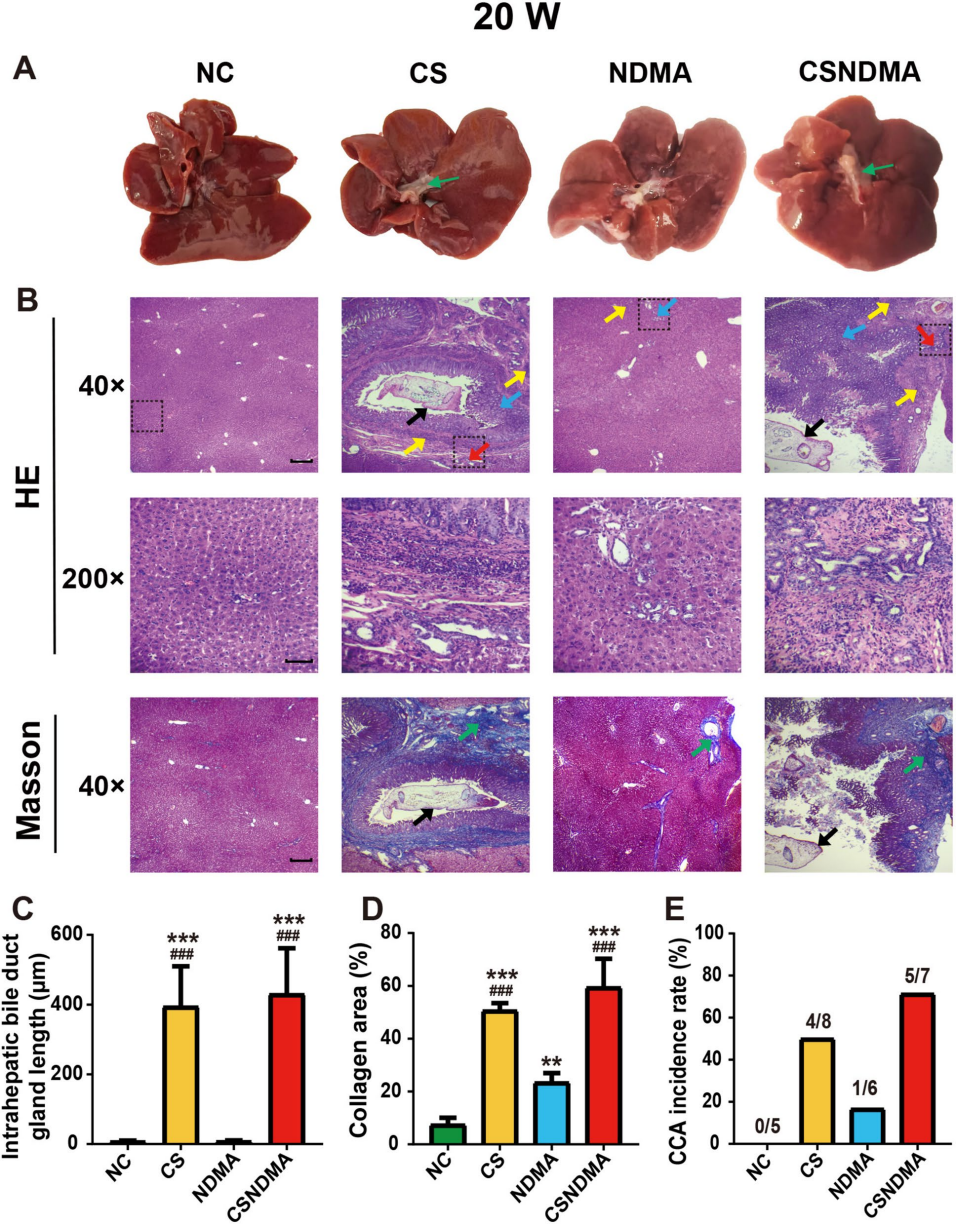
Gross appearance and histopathological changes in the livers of rats infected with *C. sinensis* at 20 weeks. **A** Gross morphology of the liver, with bile duct dilation indicated by thin green arrows. **B** H&E and Masson staining of liver sections. The 200× images correspond to the areas outlined by the dashed boxes in the 40× images. Adult worms (thick black arrows), inflammation (yellow arrows), hyperplasia (blue arrows), CCA (red arrows), and collagen fiber deposition (green arrows) are marked. **C** Intrahepatic bile duct gland length. **D** Percentage of collagen fiber area in liver tissue. **E** Incidence of CCA. Data are presented as mean ± SD. One-way ANOVA analysis of variance was used for statistical analysis. Statistical significance: ^*^P < 0.05 and ^***^P < 0.001 versus NC group; ^#^*P* < 0.05 and ^###^*P* < 0.001 versus NDMA group. Scale bars: 200 μm (40×), 50 μm (200×)

At each time point of infection, significant dilation and hypertrophy were observed in the bile ducts of rats from both the CS and CSNDMA groups (Figs. 1A, 2A, and 3A). H&E staining revealed that both groups exhibited inflammation, biliary epithelial cell hyperplasia, and adult worms of *C. sinensis* within the bile ducts. Notably, there was extensive inflammation localized to the periductal region of the common bile duct. The area of collagen deposition surrounding the bile ducts and the intrahepatic bile duct gland length in the CS and CSNDMA groups increased progressively with the duration of infection, and were significantly higher than those of the NC and NDMA groups (Figs. 1, 2, 3). Microscopic pathological analysis showed that in the CSNDMA group, CCA was first observed at 10 weeks (3/6), which gradually increased over time. In the CS group, CCA began to manifest at 17 weeks (3/6), while in the NDMA group, only one rat developed CCA at 20 weeks (1/6) (Figs. 1E, 2E, and 3E).

**Fig. 4:**
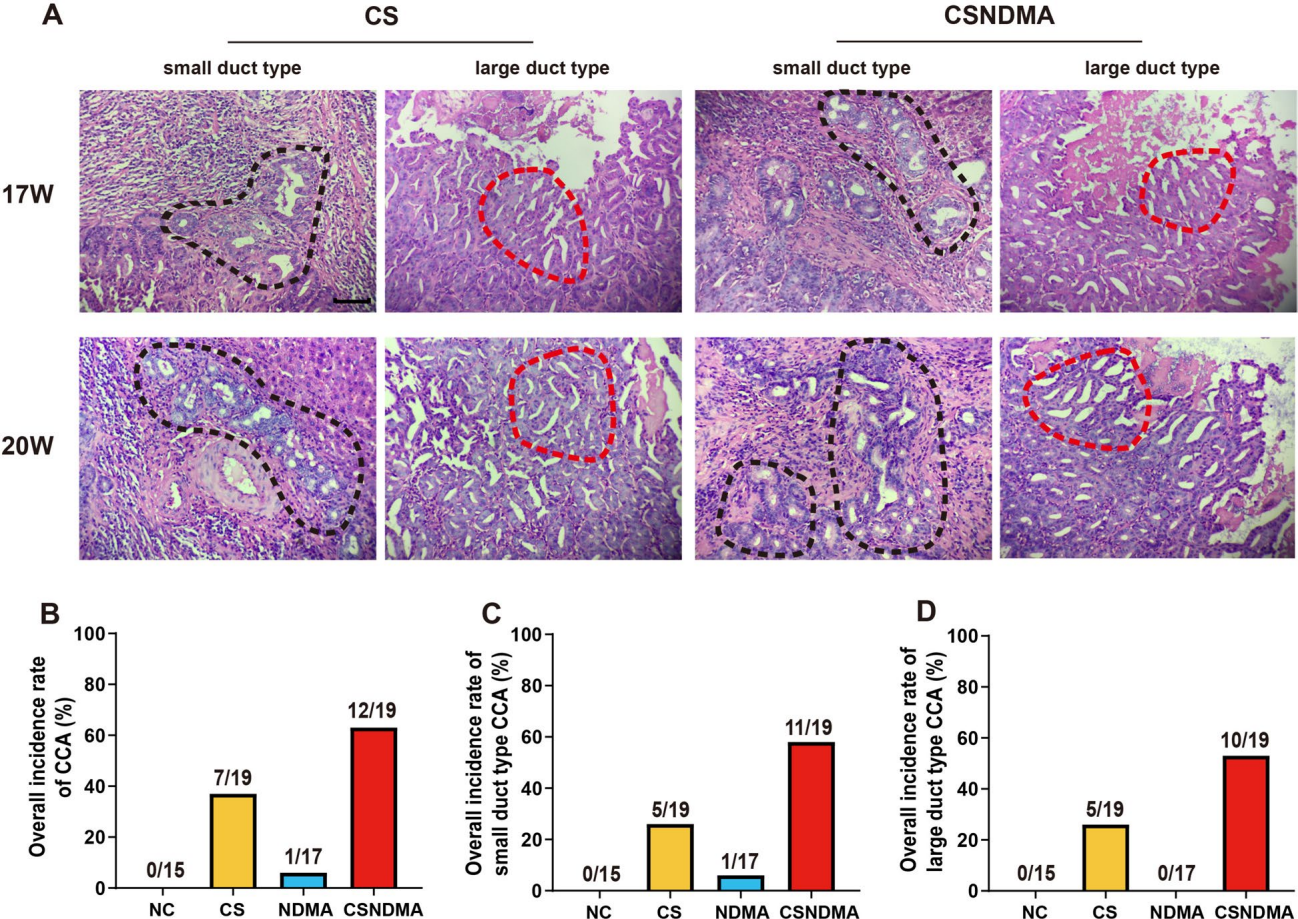
Histopathological appearance of CCA. **A** Pathological images of small- and large-duct type CCA. **B** Overall incidence rate of CCA. **C** Overall incidence rate of small-duct type CCA. **D** Overall incidence rate of large-duct type CCA. The magnification is 200× (scale bar = 50 μm), and the areas outlined by the dashed lines indicate the regions of CCA. The areas marked by the black dashed lines correspond to the small-duct type, characterized by tubular structures of varying sizes lined with low columnar to cuboidal cells, and show irregularly dilated contours. The areas marked by the red dashed lines correspond to the large-duct type, exhibiting a sieve-like configuration with dilated tumor glands featuring irregular outlines, luminal projections, and infiltrative characteristics

The typical histopathological features of CCA on H&E-stained sections included irregular tubular or acinar glands, micropapillary or cribriform structures, and infiltrative growth into the bile duct wall and hepatic parenchyma (Fig. 4A). In both the NDMA and CSNDMA groups, two rats succumbed to liver tumors or liver necrosis between weeks 16 and 20. One rat from each of the NDMA and CSNDMA groups was excluded from analysis owing to non-analyzability. The final CCA incidence rates were different across groups (Fig. 4B): 6% (1/17) in the NDMA group, 37% (7/19) in the CS group, and 63% (12/19) in the CSNDMA group. The distribution of CCA subtypes is detailed in Additional File 1: Supplementary Table S2. The CSNDMA group exhibited the highest burden of both small- and large-duct subtypes (Fig. 4C,D).

### The expression of tumor-related indicators induced by *C. sinensis* infection

**Fig. 5:**
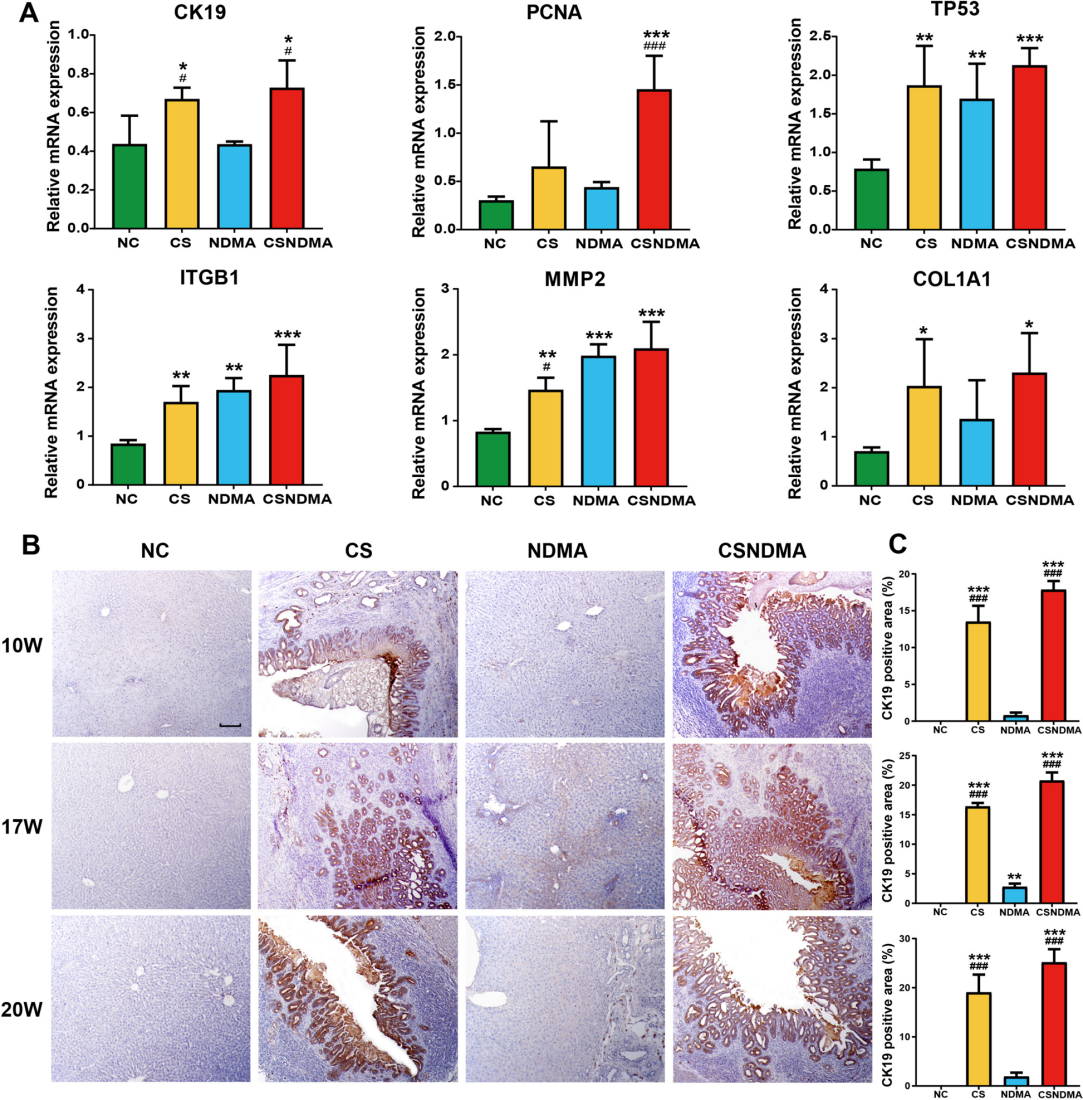
Expression levels of tumor-related genes and CK19 protein in the livers of rats induced by *C. sinensis*. **A** qPCR was conducted to assess the mRNA levels of genes involved in tumorigenesis and development in the livers of rats at 20 weeks after infection, including *CK19*, *PCNA*, *TP53*, *ITGB1*, *MMP2*, and *COL1A1*. **B** IHC staining of CK19 in the livers of rats at 10, 17, and 20 weeks post infection. The magnification is 100× (scale bar = 100 μm). **C** Statistics analysis of CK19-positive area at each time point of infection. Data are presented as mean ± SD. One-way ANOVA analysis of variance was used for statistical analysis. Statistical significance: ^*^P < 0.05, ^**^P < 0.01, and ^***^P < 0.001 versus NC group; ^#^*P* < 0.05, ^##^*P* < 0.01, and ^###^*P* < 0.001 versus NDMA group

**Fig. 6:**
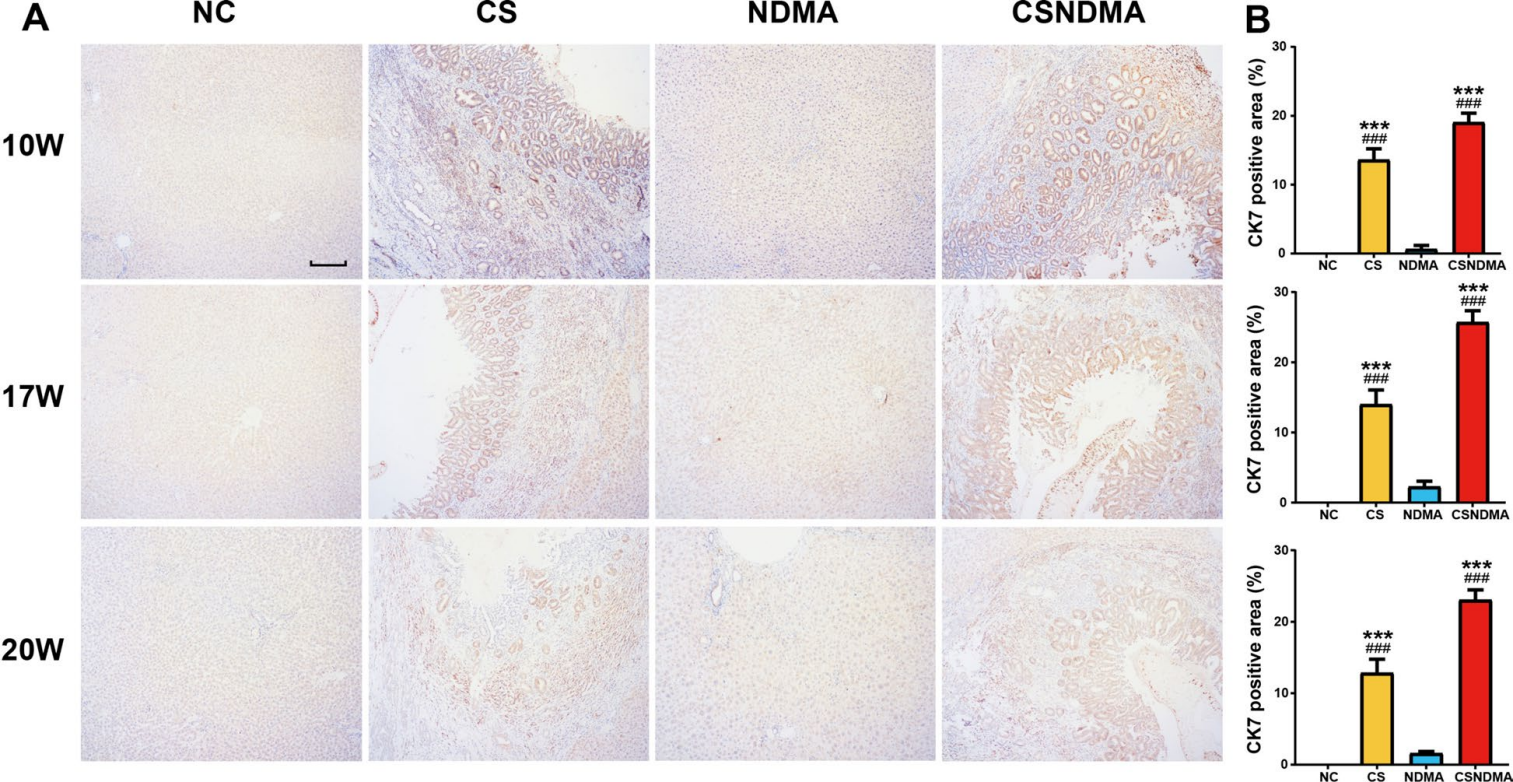
Expression levels of CK7 protein in the livers of rats following *C. sinensis* infection. **A** IHC staining of CK7 in the livers of rats at 10, 17, and 20 weeks post infection. The magnification is 100× (scale bar = 100 μm). **B** Statistics analysis of CK7-positive area at each time point of infection. Data are presented as mean ± SD. One-way ANOVA analysis of variance was used for statistical analysis. Statistical significance: ^*^P < 0.05, ^**^P < 0.01, and ^***^P < 0.001 versus NC group; ^#^*P* < 0.05, ^##^*P* < 0.01, and ^###^*P* < 0.001 versus NDMA group

**Fig. 7:**
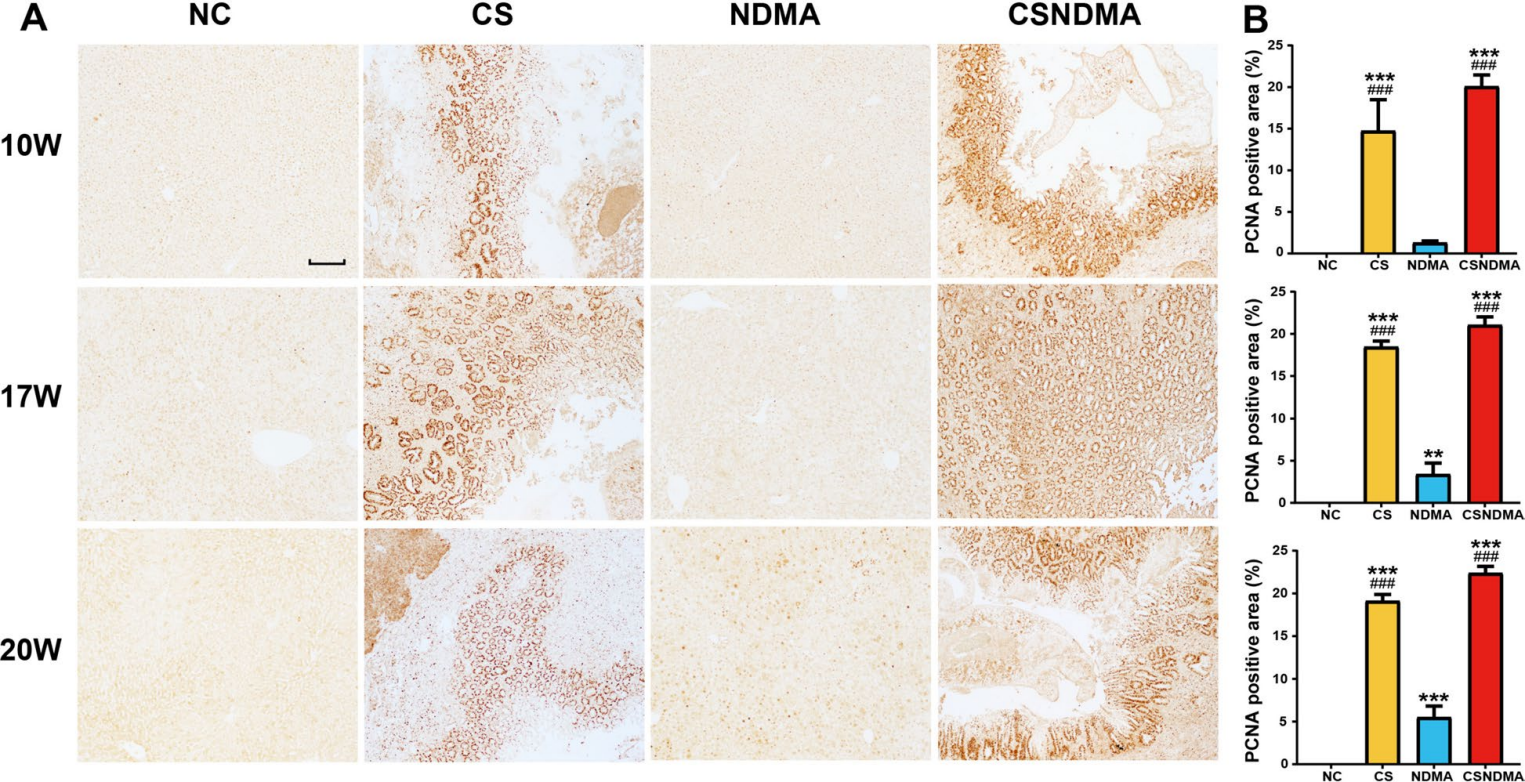
Expression levels of PCNA protein in the livers of rats following *C. sinensis* infection. **A** IHC staining of PCNA in the livers of rats at 10, 17, and 20 weeks post infection. The magnification is 100× (scale bar = 100 μm). **B** Statistics analysis of PCNA-positive area at each time point of infection. Data are presented as mean ± SD. One-way ANOVA analysis of variance was used for statistical analysis. Statistical significance: ^*^P < 0.05, ^**^P < 0.01, and ^***^P < 0.001 versus NC group; ^#^*P* < 0.05, ^##^*P* < 0.01, and ^###^*P* < 0.001 versus NDMA group

Using qPCR analysis, we observed significant upregulation of tumorigenesis-related genes, including *CK19*, *PCNA*, *TP53*, *ITGB1*, *MMP2*, and *COL1A1*, in the livers of *C. sinensis*-infected rats at 20 weeks (Fig. 5A). To further explore the oncogenic effects of *C. sinensis* on the bile ducts of rats, IHC analyses of CK19, CK7, and PCNA expression were performed. The IHC results indicated that the expression levels of CK19, CK7, and PCNA in the CS and CSNDMA groups were significantly higher than those in the NC and NDMA groups at all experimental time points. Additionally, compared with the NC group, the levels of CK19, CK7, and PCNA in the NDMA group also showed a certain degree of increase, with statistically significant differences in CK19 and PCNA levels observed after 17 weeks of infection (Figs. 5B,C, 6A,B, and 7A,B). These IHC results demonstrated that *C. sinensis* infection significantly promoted CK19 and CK7 expression in the cytoplasm of cholangiocytes, as well as PCNA expression in and around the bile ducts.

### DEGs in the livers of rats elicited by *C. sinensis* infection

**Fig. 8:**
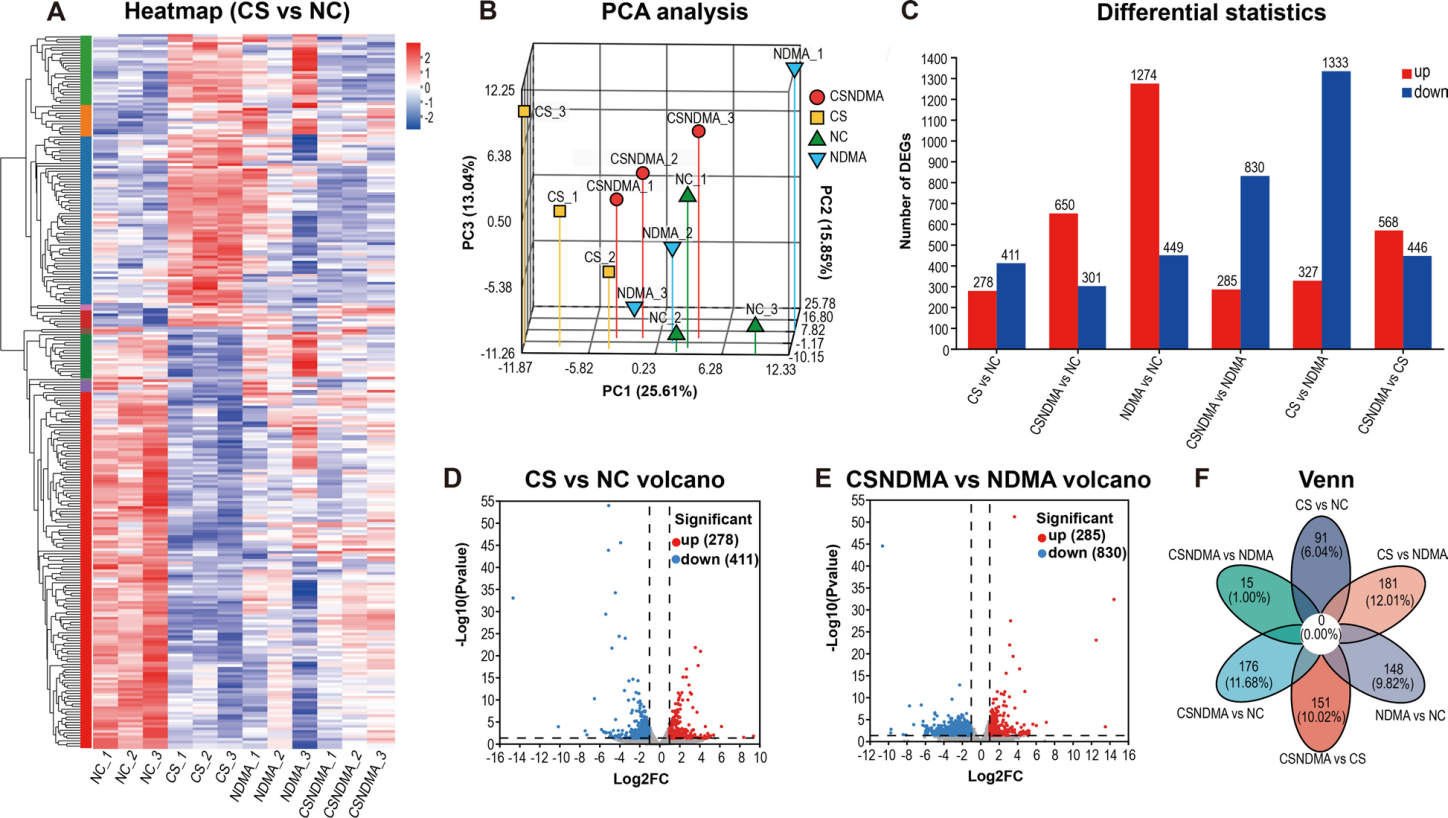
Clustering and volcano plot analysis of liver DEGs at 20 weeks post-*C. sinensis* infection. **A** Cluster heatmap of DEGs in the gene set of CS versus NC. Red and blue colors represent upregulated and downregulated genes, respectively. **B** Principal component analysis (PCA) plot of liver sample gene expression. **C** Bar chart of total DEGs. **D**, **E** Volcano plots of DEGs in the gene sets of CS versus NC and CSNDMA versus NDMA, respectively. **F** Venn diagram of DEGs in the entire gene set

To elucidate the effects of *C. sinensis* infection on rat liver at the transcriptome level, cluster analysis and differential expression quantification were performed on the DEGs. The differences in gene expression between the groups were significant, with the representative DEGs heatmap for CS versus NC shown in Fig. 8A. Principal component analysis confirmed a strong correlation among samples within the group and the distinctions between samples across different groups (Fig. 8B). The DEGs identified between the CSNDMA versus NC; CSNDMA versus CS; CSNDMA versus NDMA; CS versus NC; CS versus NDMA; and NDMA versus NC groups were 951 (650 upregulated and 301 downregulated); 1014 (568 upregulated and 446 downregulated); 1115 (285 upregulated and 830 downregulated); 689 (278 upregulated and 411 downregulated); 1660 (327 upregulated and 1333 downregulated); and 1723 (1274 upregulated and 449 downregulated), respectively (Fig. 8C–E). The qPCR test results of carcinogenic DEGs (*FADS2*, *COL4A1*, and *TUBB2A*) are displayed in Additional File 1: Supplementary Fig. S2. Venn analysis indicated no common genes among the DEGs in each gene set. The CS versus NDMA and CSNDMA versus NC gene sets exhibited the largest number of unique genes, with 181 and 176 unique genes, respectively (Fig. 8F).

### Functional enrichment analysis of liver DEGs induced by *C. sinensis* infection

**Fig. 9:**
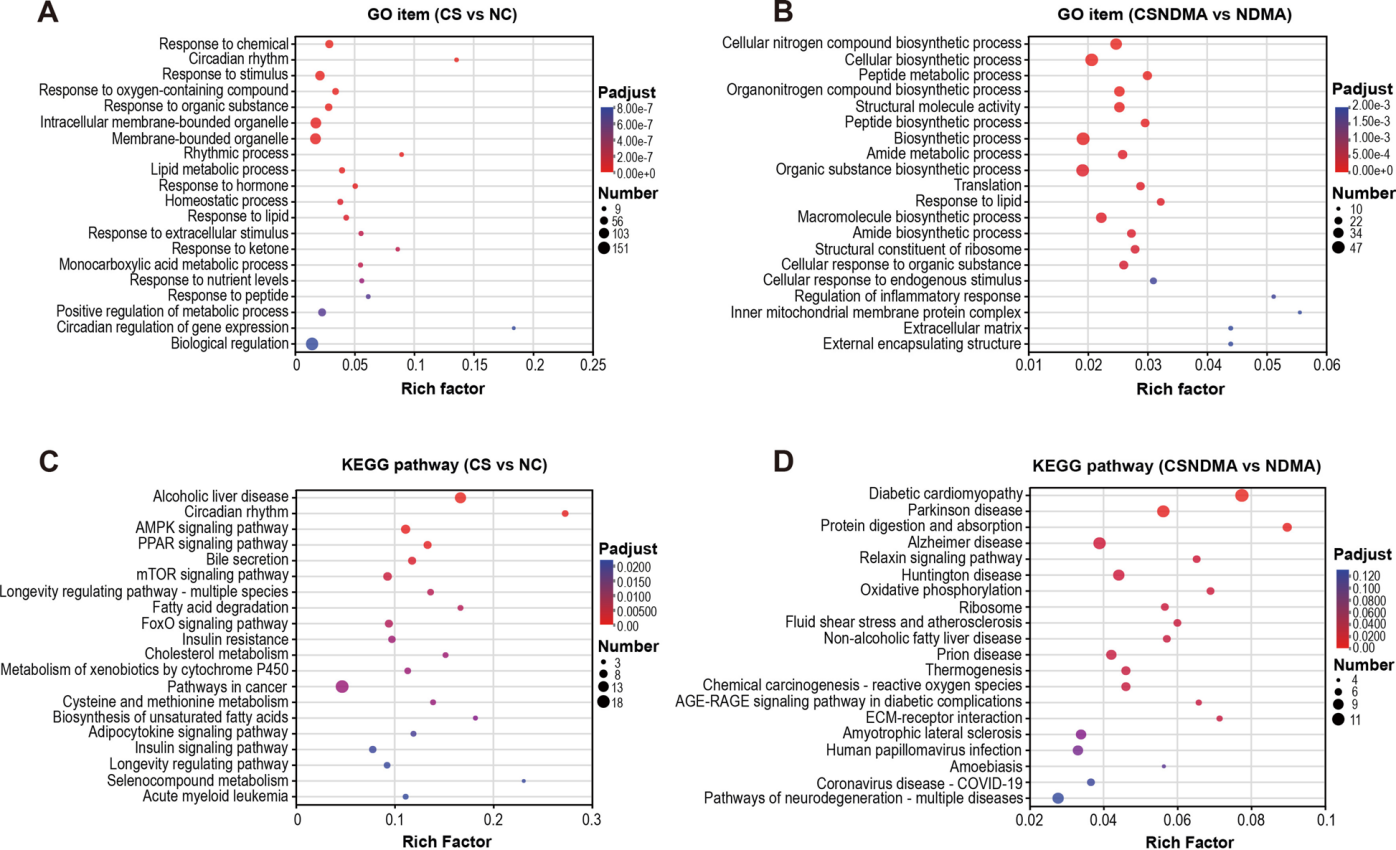
GO and KEGG enrichment analysis of DEGs in the rat livers induced by *C. sinensis* infection. **A**, **B** GO enrichment analysis of DEGs identified between the CS and NC groups and the CSNDMA and NDMA groups. **C**, **D** KEGG pathway enrichment analysis of DEGs identified between the CS and NC groups and the CSNDMA and NNDMA groups. The top 20 most enriched GO terms and KEGG pathways are displayed

Functional enrichment analyses, including GO and KEGG pathway analyses, were performed on liver DEGs. The top 20 enrichment results for each category are displayed in Fig. 9. DEGs in the CS versus NC group were predominantly enriched in GO terms related to circadian rhythm, response to oxygen-containing compounds, and lipid metabolic processes (Fig. 9A), as well as KEGG pathways, including circadian rhythm, AMPK, PPAR, mTOR, FoxO, pathways in cancer, and bile secretion (Fig. 9C). The CSNDMA versus NDMA group showed enrichment in GO terms such as biosynthetic processes of peptide and amide, translation, and peptide metabolic process (Fig. 9B), along with KEGG pathways such as oxidative phosphorylation, ribosome, chemical carcinogenesis-reactive oxygen species, AGE–RAGE signaling pathway in diabetic complications, and extracellular matrix (ECM)-receptor interaction (Fig. 9D). The enrichment analysis results for CS versus NDMA, CSNDMA versus CS, NDMA versus NC, and CSNDMA versus NC are presented in Additional File 1: Supplementary Figs. S3A–D and S4A–D, respectively.

## Discussion

Numerous studies have demonstrated that the Syrian golden hamster is a relatively suitable model animal for investigating *C. sinensis*-mediated CCA, whereas no definite tumorigenic changes have been observed in BALB/c, C3H/He, ICR, and other mouse strains infected with *C. sinensis* and simultaneously exposed to *N*-nitrosodimethylamine [4, 17, 28, 29]. However, there is a lack of research on the occurrence of CCA in other definitive hosts that are more conducive to the survival of *C. sinensis*, such as rats and rabbits. Sprague–Dawley rats are commonly used as animal models for collecting adult worms of *C. sinensis* and studying the pathogenic mechanisms of infection [30–32]. In our study, the incidence of CCA in the group infected solely with *C. sinensis* was 37%, whereas Uddin et al. reported no incidence of CCA in the simple infection group using golden hamsters [17]. This discrepancy may be attributed to the fact that rats serve as more suitable hosts for *C. sinensis*, similar to humans. Thus, the rat model, with its advantages of low cost, ease of handling, and enabling long-term stable survival and isolation of adult worms, is ideal for studying the pathogenesis and mechanisms of CCA caused by chronic *C. sinensis* infection.

As observed in our previous animal studies and other reports [16, 33–35], this study also found inflammation and hyperplasia of bile duct epithelial cells in the livers of rats from both the CS and CSNDMA groups. Furthermore, consistent with the findings of Jeong et al., liver pathology in the *C. sinensis* infection group and the combination treatment group with *N*-nitrosodimethylamine was more severe than that in the *N*-nitrosodimethylamine-only group, with lesions originating in the bile ducts and progressively worsening [36]. As reported by Qi et al. [32], our results confirm that *C. sinensis* infection could indeed significantly promote collagen deposition in the liver of rats, leading to the development of periductal fibrosis and liver fibrosis. It has been well-documented that collagen fibers are prominent in various solid tumors, such as squamous cell carcinoma and colon cancer [37, 38], and they are also a remarkable feature of CCA [39]. Periductal fibrosis, characterized by extracellular matrix remodeling and inflammation, could promote biliary epithelial cell proliferation, DNA damage, and carcinogenesis, facilitating the development of CCA [40, 41]. Overall, our findings suggest that *C. sinensis* infection primarily induces pathological damage to the liver, particularly targeting the bile ducts, and these cumulative pathological changes may collectively contribute to the progression of CCA.

During tumorigenesis, abnormal expression or mutations of multiple tumor-related genes occur. Our qPCR results showed that *C. sinensis* infection significantly elevated the levels of *CK19*, *PCNA*, *TP53*, *ITGB1*, *MMP2*, and *COL1A1* in rat liver tissue, particularly in the CSNDMA group. These genes are crucial in the progression of CCA [17, 42–46]. The *TP53* gene, a major tumor suppressor, is mutated in approximately 50% of tumors [47]. The integrin β1 encoded by *ITGB1* facilitates cell adhesion, migration, and signaling, thereby promoting tumor growth [48]. *MMP2*, a matrix metalloproteinase, contributes to ECM degradation, enhancing tumor invasion and metastasis [45]. Collagen, encoded by *COL1A1*, is a vital ECM component, and its abnormal deposition is closely linked to tumor invasion and metastasis [46]. IHC analyses revealed that the protein levels of CK19, CK7, and PCNA were significantly higher in the CS and CSNDMA groups compared with the NC and NDMA groups. CK19, a cytokeratin expressed in epithelial cells, serves as a key immunohistochemical marker for iCCA, aiding in its early detection and differentiation from hepatocellular carcinoma (HCC) [42, 49]. Overexpression of CK19 has also been reported in *C. sinensis* + diethylnitrosamine-induced rat livers [32] and *O. viverrini*-induced hamster CCA model [50]. CK7, a type II intermediate filament protein expressed in glandular and transitional epithelia, serves as another critical immunohistochemical marker for CCA [8]. Both CK19 and CK7 exhibit high sensitivity for identifying biliary tract origin neoplasms, and their combined detection may serve as a potential prognostic indicator for CCA [51, 52]. PCNA, a cell-cycle-related protein crucial for DNA replication in cancer cells, exhibited high expression in the liver tissue, particularly in the bile ducts of CS + NDMA gold hamsters [17, 53]. Collectively, these findings suggest that *C. sinensis* infection may enhance the expression of oncogenic genes and upregulate CCA-specific marker (CK19, CK7) as well as the malignant proliferation marker PCNA in rat liver, particularly around the bile duct, which could potentially contribute to CCA initiation or progression.

Our transcriptome analysis of the liver at 20 weeks post infection indicated that *C. sinensis* infection induces abundant DEGs and notable intergroup variations. KEGG enrichment analysis revealed that *C. sinensis* infection enriches pathways associated with tumorigenesis, metabolic reprogramming, and inflammation in the liver of rats, such as pathways of AMPK, PPAR, mTOR, and FoxO. AMPK is essential for maintaining energy and metabolic balance, influencing processes such as lipogenesis, glycolysis, and tricarboxylic acid (TCA) cycle; its abnormalities are closely related to cancer and metabolic disorders [54, 55]. PPAR is a key metabolic regulator of lipid metabolism and inflammation in the liver, with abnormalities leading to hepatic steatosis, steatohepatitis, fatty fibrosis, and liver cancer [56, 57]. The mTOR pathway is a critical regulatory factor for cellular metabolism, growth, and survival, and is involved in the carcinogenesis and progression of biliary tract cancer and HCC [58, 59]. As a pivotal signaling hub, mTOR modulates inflammatory responses by regulating cellular metabolism and autophagy; its hyperactivation may promote chronic inflammation and tumor progression [60–62]. Additionally, abnormal expression of FoxO is associated with metabolic diseases, cancer, and reduced lifespan [63]. Meanwhile, in the CS versus NC and CSNDMA versus NDMA groups, significant enrichment of pathways such as insulin resistance, diabetic cardiomyopathy, and degenerative neurological diseases, including Parkinson’s, Alzheimer’s, and Huntington’s, were observed, echoing findings from our previous omics analysis of serum from patients with HCC infected with *C. sinensis* [64].

Moreover, our combined GO and KEGG analyses revealed that *C. sinensis* infection may significantly disrupt the circadian rhythm in rat liver. Our earlier research indicated that the early stages of *C. sinensis* infection notably alter the gut rhythm in mice [65]. The circadian rhythm is crucial for maintaining liver homeostasis, regulating energy metabolism, xenobiotic metabolism, and immune responses. Disruption of this rhythm can accelerate liver diseases such as fatty liver, cirrhosis, hepatitis, and liver cancer, which in turn can further perturb circadian function [66–68]. To meet the heightened energy demands of rapid tumor cell proliferation, invasion, and metastasis, metabolic reprogramming is a hallmark of cancer, including CCA [69, 70]. Our GO enrichment analysis confirmed that *C. sinensis* infection or its interaction with *N*-nitrosodimethylamine may primarily impact biosynthesis and substance metabolism processes in rat liver, including lipid response, organonitrogen compound biosynthesis, and lipid and amide metabolism. KEGG analysis further indicated significant enrichment in pathways related to fatty acid degradation, unsaturated fatty acid biosynthesis, as well as cysteine and methionine metabolism. Dysregulation of lipid metabolism is one of the most pronounced metabolic changes in cancer, significantly influencing cancer progression and treatment responses by remodeling the tumor microenvironment [71, 72]. These findings suggest that circadian rhythm disruption and alterations in metabolism and biosynthesis triggered by *C. sinensis*, particularly lipid metabolism reprogramming, may contribute to CCA progression.

## Conclusions

**Fig. 10:**
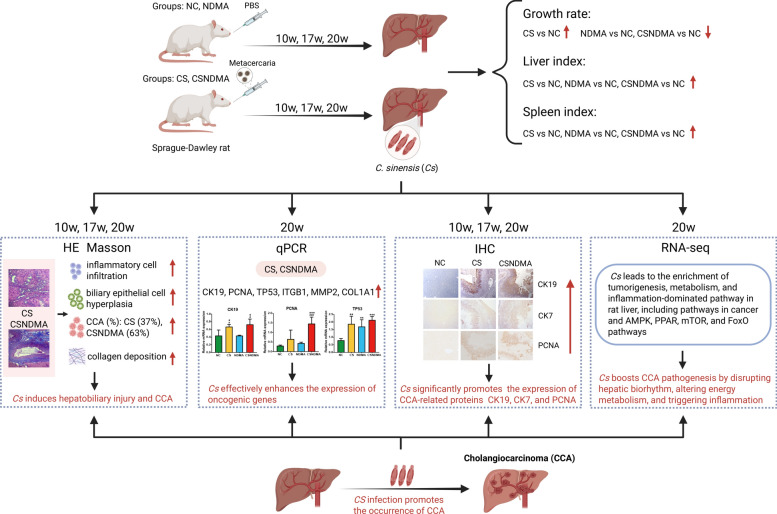
*C. sinensis* infection promotes the occurrence of CCA in rats. The top left of the figure shows the experimental design and the top right shows the gross body composition metrics. The middle panels summarize the histochemistry, gene expression analysis, and transcriptome, respectively. At the bottom of the figure, there is a summary of the occurrence of CCA caused by infection with *C. sinensis*. Created with BioRender.com

This study successfully established a rat model of *C. sinensis*-associated CCA, demonstrating that infection may potentially promote CCA progression. The infection triggered bile duct-centric liver pathology and fibrosis, accompanied by the upregulation of oncogenic markers (e.g., *CK19*, *CK7*, *PCNA*, and *TP53*). Transcriptomic analysis revealed activation of pathways related to tumorigenesis, metabolic reprogramming, and inflammation, including the AMPK, PPAR, mTOR, and FoxO pathways. Notably, *C. sinensis* infection disrupted hepatic circadian rhythm and reprogrammed lipid metabolism, which may be critical for tumorigenesis. However, the specific molecular mechanisms underlying these changes remain unclear and warrant further investigation. Collectively, these findings indicate that *C. sinensis* could serve as a key driver of CCA progression through complex regulatory mechanisms (Fig. 10), providing a foundation for future mechanistic and therapeutic research.

## Supplementary Information

Additional file 1: Table S1. The primer sequences used in this experiment. Supplementary Fig. S1. Effects of *C. sinensis* infection on body weight and hepato-splenic indices of rats. Table S2. The incidence case of small and large-duct type CCA in rats. Supplementary Fig. S2. Expression levels of tumor-related genes. Supplementary Fig. S3. GO enrichment analysis of DEGs in the rat livers induced by *C. sinensis* infection. Supplementary Fig. S4. KEGG pathway enrichment analysis of DEGs in the rat livers induced by *C. sinensis* infection.

## Data Availability

The datasets presented in this study can be found in online repositories. The raw data have been deposited in the NCBI database under accession no. PRJNA1253197.

## Abbreviations

*C. sinensis*: *Clonorchis sinensis*
CCA: Cholangiocarcinoma
*O. viverrini*: *Opisthorchis viverrini*
PBS: Phosphate-buffered saline
NC: Negative control
CS: *C. sinensis* infection
NDMA: *N*-nitrosodimethylamine induction
CSNDMA: *N*-nitrosodimethylamine-induced and *C. sinensis* infection
H&E: Hematoxylin and eosin
iCCA: Intrahepatic cholangiocarcinoma
qPCR: Quantitative real‑time PCR
PCNA: Proliferating cell nuclear antigen
CK19: Cytokeratin 19
CK7: Cytokeratin 7
IHC: Immunohistochemistry
DEGs: Differentially expressed genes
GO: Gene Ontology
KEGG: Kyoto Encyclopedia of Genes and Genomes
SD: Standard deviation
ECM: Extracellular matrix
