# Supplementary material for: Exploratory study on the role of Clonorchis sinensis infection in promoting cholangiocarcinoma progression
Source: Parasit Vectors. 2025 Dec 9;19:29. doi: 10.1186/s13071-025-07183-2 (PMC12801685; doi:10.1186/s13071-025-07183-2)
Supplement: Supplementary file 1 — Additional file 1: Table S1. The primer sequences used in this experiment. Supplementary Fig. S1. Effects of C. sinensis infection on body weight and hepato-splenic indices of rats. Table S2. The incidence case of small and large-duct type CCA in rats. Supplementary Fig. S2. Expression levels of tumor-related genes. Supplementary Fig. S3. GO enrichment analysis of DEGs in the rat livers induced by C. sinensis infection. Supplementary Fig. S4. KEGG pathway enrichment analysis of DEGs in the rat livers induced by C. sinensis infection. [file 13071_2025_7183_MOESM1_ESM.docx]

**Table S1.** The primer sequences used in this experiment.

| Primer | Sequence (5′ to 3′) | Base number |
| --- | --- | --- |
| PCNA-F | GATGTTCCTCTCGTTGTGGAG | 21 |
| PCNA-R | CATTGCAGTTAAGAGCCTTCC | 21 |
| CK19-F | AGTCTTCTCAGCCAAACCCTC | 21 |
| CK19-R | CTGGTCTGTGGAACTAGGCA | 20 |
| TP53-F | CCCCACCGCCTGTAAGATTC | 20 |
| TP53-R | GAGGGGTGGGGGATGGATA | 19 |
| ITGB1-F | GACACTGCTGGTGCTAATGC | 20 |
| ITGB1-R | TTCCGCACGCATCATTGAGA | 20 |
| MMP2-F | GCCCAGAGACTGCTATGTCC | 20 |
| MMP2-R | GCTGGTGCAGCTCTCATACT | 20 |
| COL1A1-F | CATGAGCCGAAGCTAACCCC | 20 |
| COL1A1-R | CAGGTTTCCACGTCTCACCA | 20 |
| FADS2-F | TTGCACAAGATTGCCCCA | 18 |
| FADS2-R | GGCTTCTCTTGGTATTCAATGCC | 23 |
| COL4A1-F | TGCCAAGCACGGAAGAGAATG | 21 |
| COL4A1-R | GTACCCAGCTATTAAGGCTGCT | 22 |
| TUBB2A-F | TTGTGTTCGGTCAGAGTGGT | 20 |
| TUBB2A-R | GACTCCTTCCTCACCACATC | 20 |
| β-actin-F | CGTTGACATCCGTAAAGAC | 19 |
| β-actin-R | TAGGAGCCAGGGCAGTA | 17 |


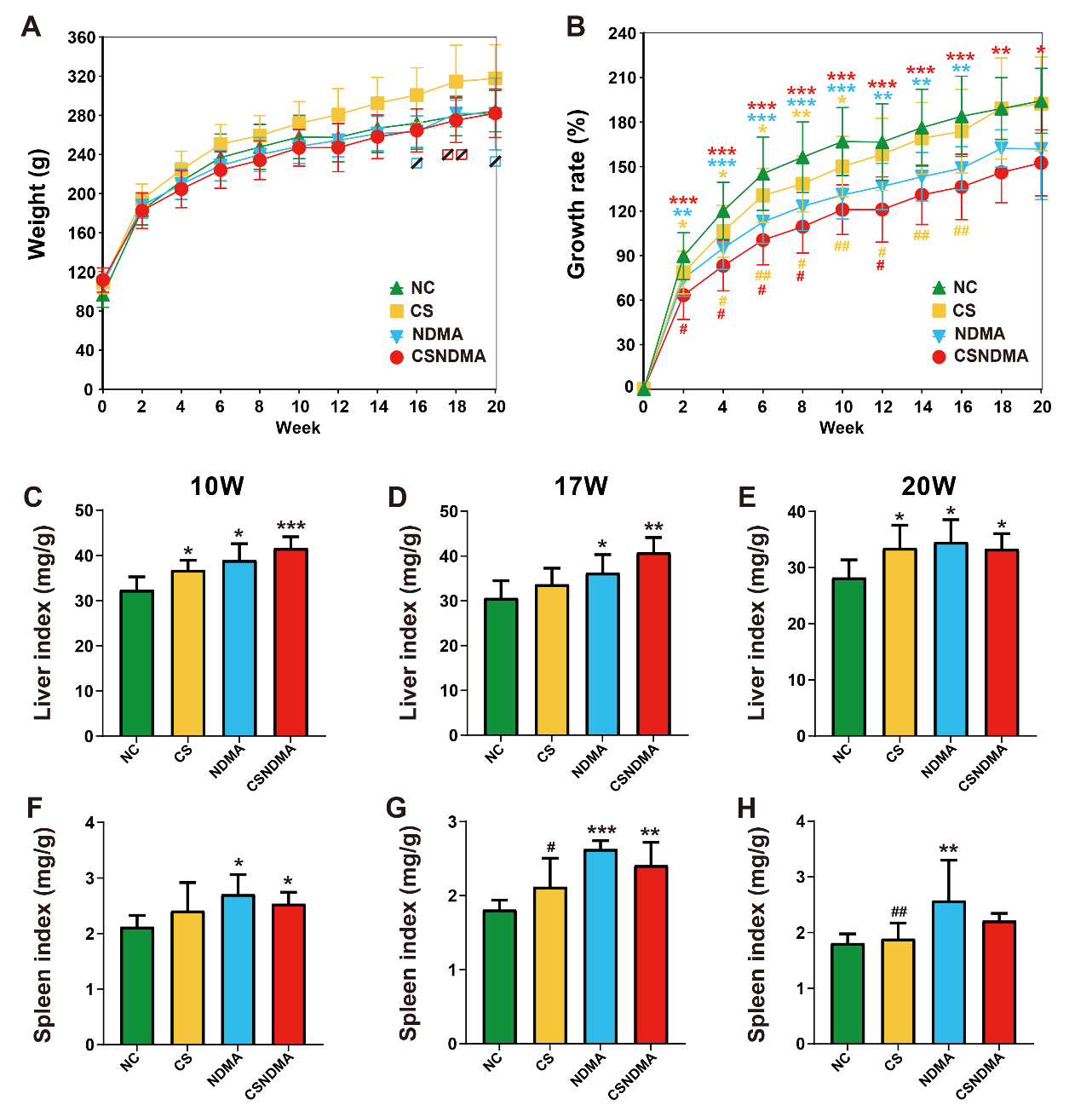


**Fig. S1.** Effects of *C. sinensis* infection on body weight and hepato-splenic indices of rats. **A** Body weight change curve of rats. **B** Body weight growth rate change curve of rats relative to the average body weight of each group of rats at the beginning of the experiment. The hepatic/splenic index is calculated by the following formula: hepatic/splenic wet weight (mg)/rat body weight (g). **C-E** Hepatic index of rats at 10 weeks (**C**), 17 weeks (**D**), and 20 weeks (**E**), respectively. **F-H** Splenic index of rats at 10 weeks (**F**), 17 weeks (**G**), and 20 weeks (**H**), respectively. Data are presented as mean ± SD. Use one-way ANOVA analysis of variance for statistical analysis. Statistical significance: ^*^ *P* < 0.05, ^**^ *P* < 0.01, and ^***^ *P* < 0.001 versus NC group; ^#^ *P* < 0.05, ^##^ *P* < 0.01, and ^###^ *P* < 0.001 versus NDMA group

**Table S2.** The incidence case of small and large duct type CCA in rats.

| Group | No. of case | No. of small duct type CCA case | No. of large duct type CCA case |
| --- | --- | --- | --- |
| 10wNC | 5 | 0 | 0 |
| 10wCS | 5 | 0 | 0 |
| 10wNDMA | 5 | 0 | 0 |
| 10wCSNDMA | 6 | 2 | 3 |
| 17wNC | 5 | 0 | 0 |
| 17wCS | 6 | 1 | 3 |
| 17wNDMA | 6 | 0 | 0 |
| 17wCSNDMA | 6 | 4 | 4 |
| 20wNC | 5 | 0 | 0 |
| 20wCS | 8 | 4 | 2 |
| 20wNDMA | 6 | 1 | 0 |
| 20wCSNDMA | 7 | 5 | 3 |


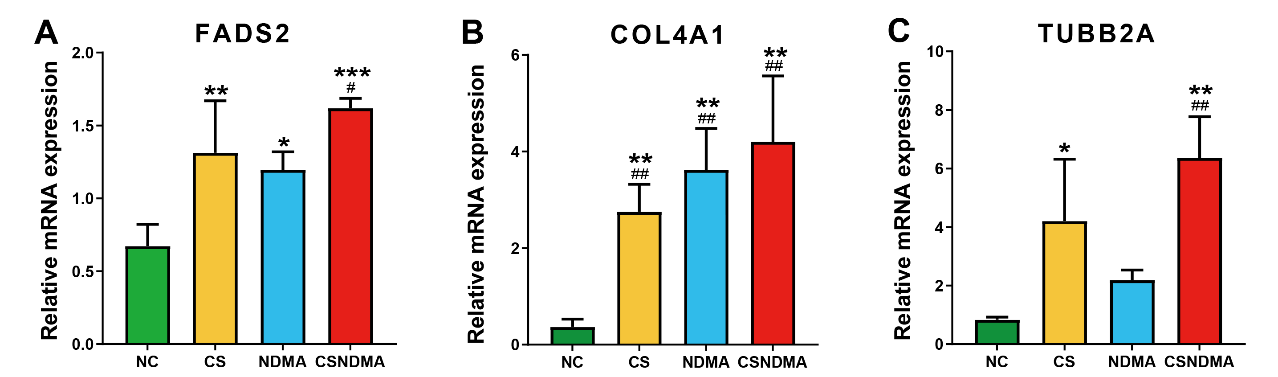


**Fig. S2.** Expression levels of tumor-related genes. **A** The mRNA level of FADS2 in the liver of rats at 20 weeks post-infection. **B** The mRNA level of COL4A1 in the liver of rats at 20 weeks post-infection. **C** The mRNA level of TUBB2A in the liver of rats at 20 weeks post-infection. Data are presented as mean ± SD. Use one-way ANOVA analysis of variance for statistical analysis. Statistical significance: ^*^ *P* < 0.05, ^**^ *P* < 0.01, and ^***^ *P* < 0.001 versus NC group; ^#^ *P* < 0.05, ^##^ *P* < 0.01, and ^###^ *P* < 0.001 versus NDMA group


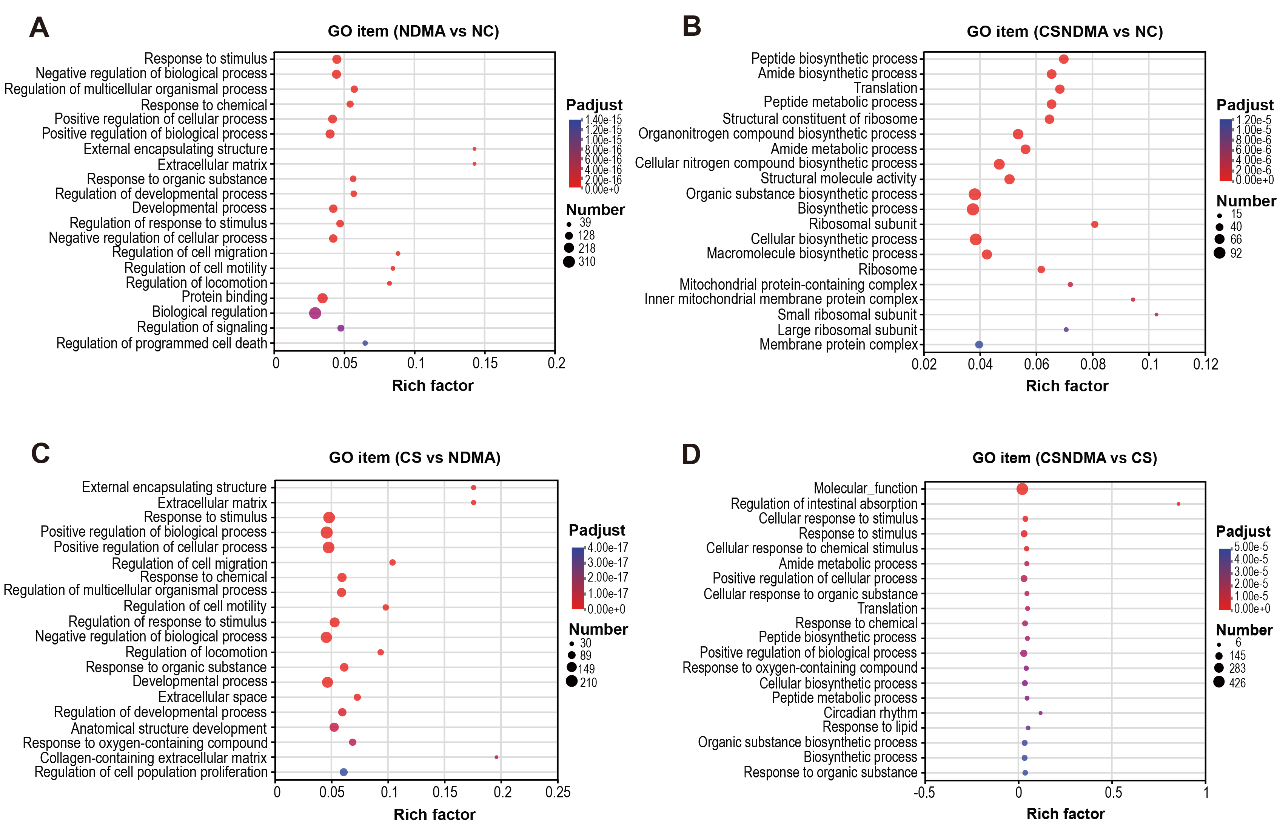


**Fig. S3.** GO enrichment analysis of DEGs in the rat livers induced by *C. sinensis* infection. **A-D** GO enrichment analysis of DEGs identified between the NDMA and NC groups, the CSNDMA and NC groups, the CS and NDMA groups, and the CSNDMA and CS groups. The top 20 most enriched GO terms are displayed


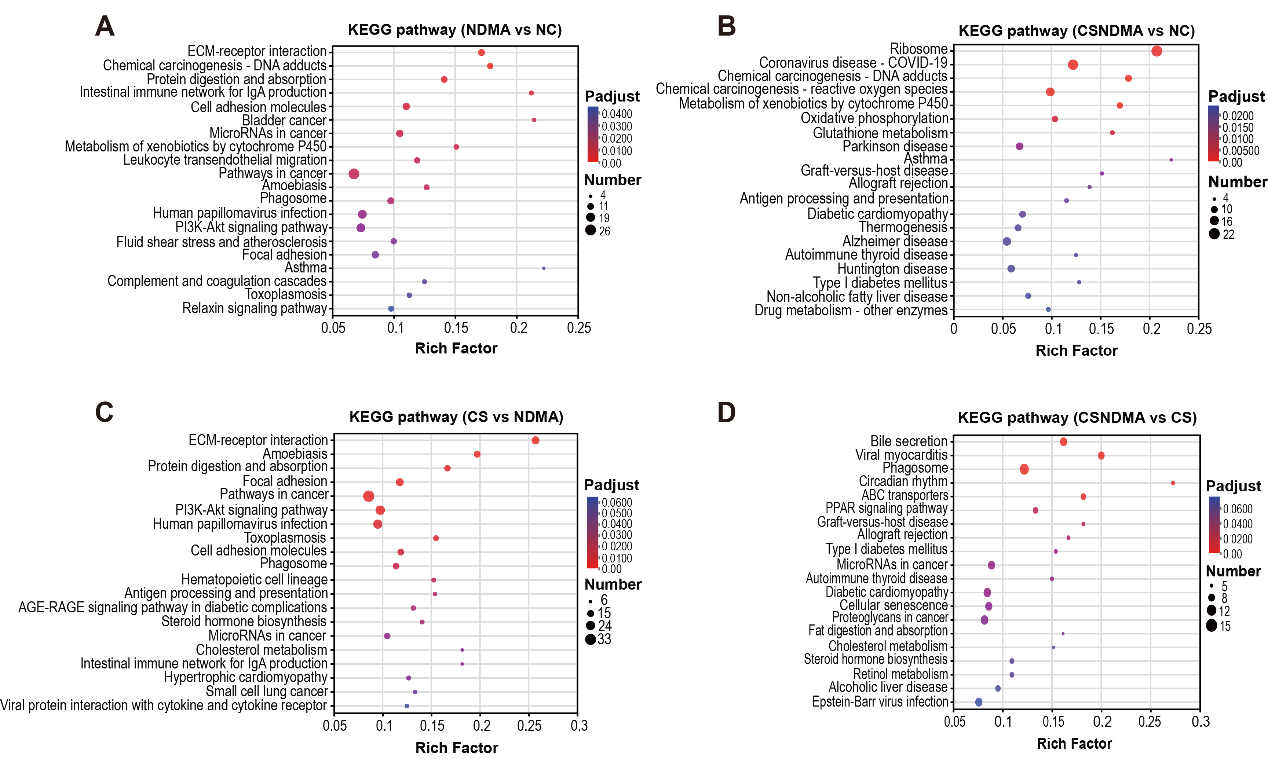


**Fig. S4.** KEGG pathway enrichment analysis of DEGs in the rat livers induced by *C. sinensis* infection. **A-D** KEGG pathway enrichment analysis of DEGs identified between the NDMA and NC groups, the CSNDMA and NC groups, the CS and NDMA groups, and the CSNDMA and CS groups. The top 20 most enriched pathways are presented
